# Correction to “Construction of the Adjusted Scoliosis 3D Finite Element Model and Biomechanical Analysis under Gravity”

**DOI:** 10.1111/os.70317

**Published:** 2026-04-13

J. Li , Z. An , J. Wu , et al., “Construction of the Adjusted Scoliosis 3D Finite Element Model and Biomechanical Analysis under Gravity,” Orthopaedic Surgery 15 (2023): 606–616, 10.1111/os.13572.36482875 PMC9891986

In Figure 7, the subgraph labeled T2 incorrectly uses the subgraph that originally belonged to L2, resulting in inconsistency between the image and the annotations. The subgraph T2 has been replaced with the accurate version. All the images and annotations under this figure (including T2 and L2) are now accurate.

The correct version is shown below:

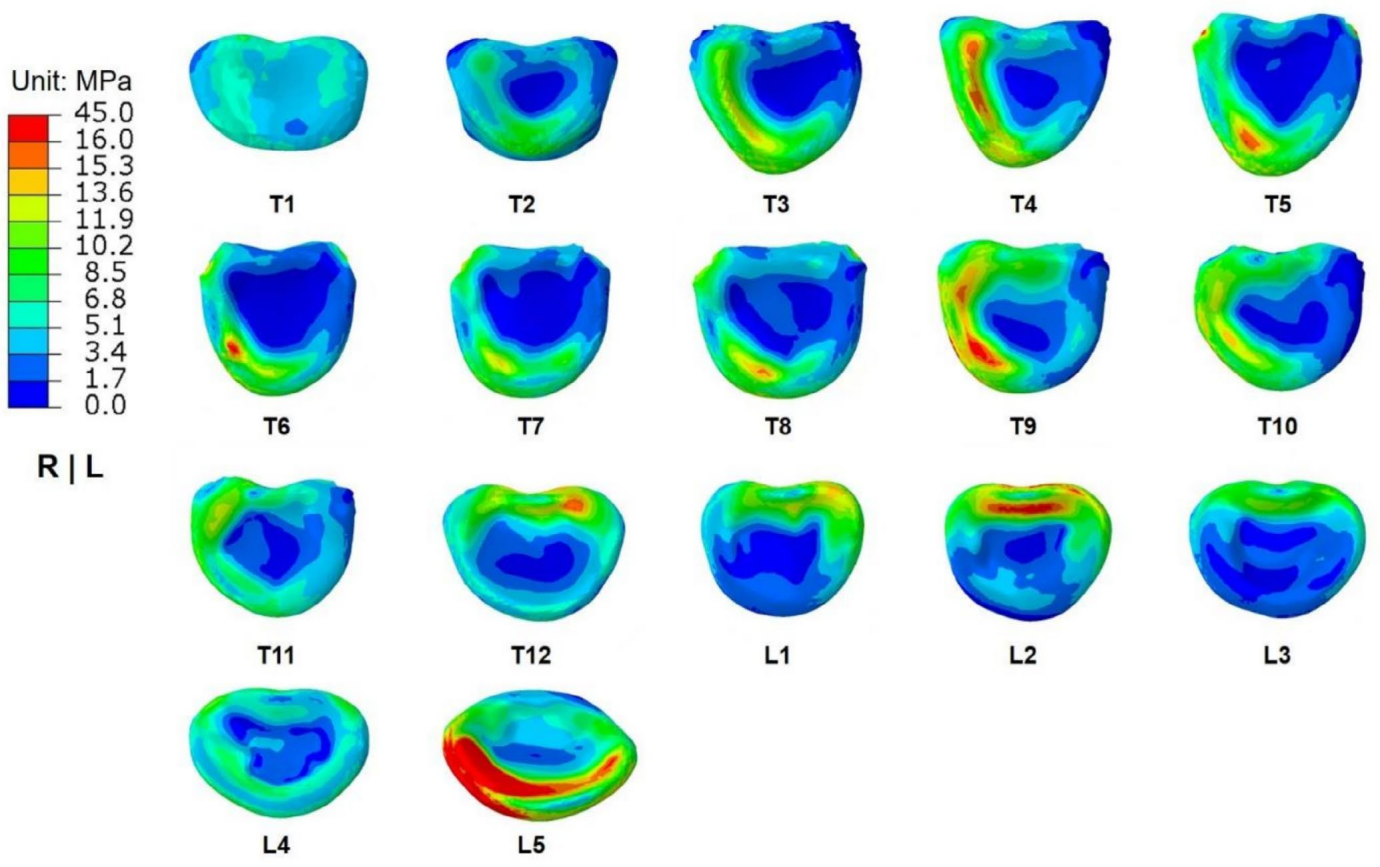

We apologize for this error.

